# Vitamin Variation in Capsicum Spp. Provides Opportunities to Improve Nutritional Value of Human Diets

**DOI:** 10.1371/journal.pone.0161464

**Published:** 2016-08-17

**Authors:** Michael B. Kantar, Justin E. Anderson, Sarah A. Lucht, Kristin Mercer, Vivian Bernau, Kyle A. Case, Nina C. Le, Matthew K. Frederiksen, Haley C. DeKeyser, Zen-Zi Wong, Jennifer C. Hastings, David J. Baumler

**Affiliations:** 1 Department of Tropical Plant & Soil Sciences, University of Hawaii at Manoa, Honolulu, HI, United States of America; 2 Department of Epidemiology, Harvard T.H. Chan School of Public Health, Harvard University, Boston, MA, United States of America; 3 Department of Horticulture and Crop Science, The Ohio State University, Columbus, OH, United States of America; 4 Department of Food Science and Nutrition, University of Minnesota-Twin Cities, St. Paul, MN, United States of America; 5 Microbial and Plant Genome Institute, University of Minnesota-Twin Cities, St. Paul, MN, United States of America; 6 Biotechnology Institute, University of Minnesota-Twin Cities, St. Paul, MN, United States of America; State University of Rio de Janeiro, BRAZIL

## Abstract

Chile peppers, native to the Americas, have spread around the world and have been integrated into the diets of many cultures. Much like their heat content, nutritional content can vary dramatically between different pepper types. In this study, a diverse set of chile pepper types were examined for nutrient content. Some pepper types were found to have high levels of vitamin A, vitamin C, or folate. Correlations between nutrient content, species, cultivation status, or geographic region were limited. Varietal selection or plant breeding offer tools to augment nutrient content in peppers. Integration of nutrient rich pepper types into diets that already include peppers could help combat nutrient deficiencies by providing a significant portion of recommended daily nutrients.

## Introduction

The genus *Capsicum* contains five domesticated species: *Capsicum annuum*, *Capsicum chinense*, *Capsicum frutescens*, *Capsicum baccatum*, and *Capsicum pubescens*, all commonly known as peppers. Of these species, *C*. *annuum* is the most economically important [1,2]. Domesticated between six and nine thousand years ago in what is modern day Mexico [2,3], peppers are now eaten daily by almost a quarter of the world’s population as a common ingredient in European, Asian, African, and American cuisines [1,4].

Ongoing selection and improvement of the domesticated types have resulted in variation within and among species for fruit color, shape [5,6], secondary metabolites [7], and disease resistance [8]. Historically used in Mayan medicinal practices [9], recent studies have explored the potential antimicrobial properties [10], as well as the health benefits of consuming phytochemical compounds found in peppers [7].

Peppers are consumed raw, cooked, and as a spice [4]. The secondary metabolites commonly connected with peppers are capsaicinoids, the compounds that produce their “heat”. Peppers are also a good source of vitamin C, vitamin A, vitamin E, and folate [11,12,13]. A number of factors can affect their nutritional content including agronomics [14], harvest time [15], storage and preparation technique [16,17,18], and cultivar type [13,19,20,21].

Understanding and improving the nutritional content in pepper varieties could aid in prevention and treatment strategies for malnutrition. Nutritional deficiencies, and their attending diseases, remain prevalent in both the developed and developing world [22,23,24]. Capsaicin’s thermogenic effect could help in weight loss [25] and maintaining weight reduction [26], an important component in addressing the current obesity epidemic in the developed world [27]. Vitamin A, found in peppers, is an essential nutrient required to maintain healthy eyesight and a functional immune system [28,29]. Vitamin A deficiency affects over 125 million children under the age of five, with the most severe symptom being blindness estimated to affect 250,000 to 500,000 children annually [30,31,32]. Vitamin C, a nutrient and antioxidant, is present in some pepper varieties at twice the concentration as in tomatoes, apples, or oranges per gram of fruit weight [12]. Folate, an important B-group vitamin known to reduce risk of cardiovascular disease and cancer, is also present to varying degrees in pepper varieties [11]. Insufficient folate intake can affect proper fetal and infant brain development [24,33,34,35], therefore many countries mandate the fortification of food with folic acid [36]. Identifying varieties of peppers that have high levels of multiple nutrients would be most effective at serving to enhance the diet, as foods that contain high levels of multiple vitamins can be more broadly beneficial by reducing requirements for extra biofortification [33].

Peppers, with their high nutritional content and global consumption, may have a role to play in reducing nutrient deficiencies. In this study, a diverse collection of diverse peppers was evaluated for vitamin A, vitamin C, folate, and capsaicin content. Relationships were explored among nutrient levels, geographic origin, species, and breeding status (heirloom/landrace or modern cultivar). Pepper types were identified with high nutrient content and a range of Scoville heat levels, suggesting that subsequent breeding could develop nutrient-packed mild or hot peppers.

## Materials and Methods

### Assembly and propagation of diverse germplasm

**Fig 1:**
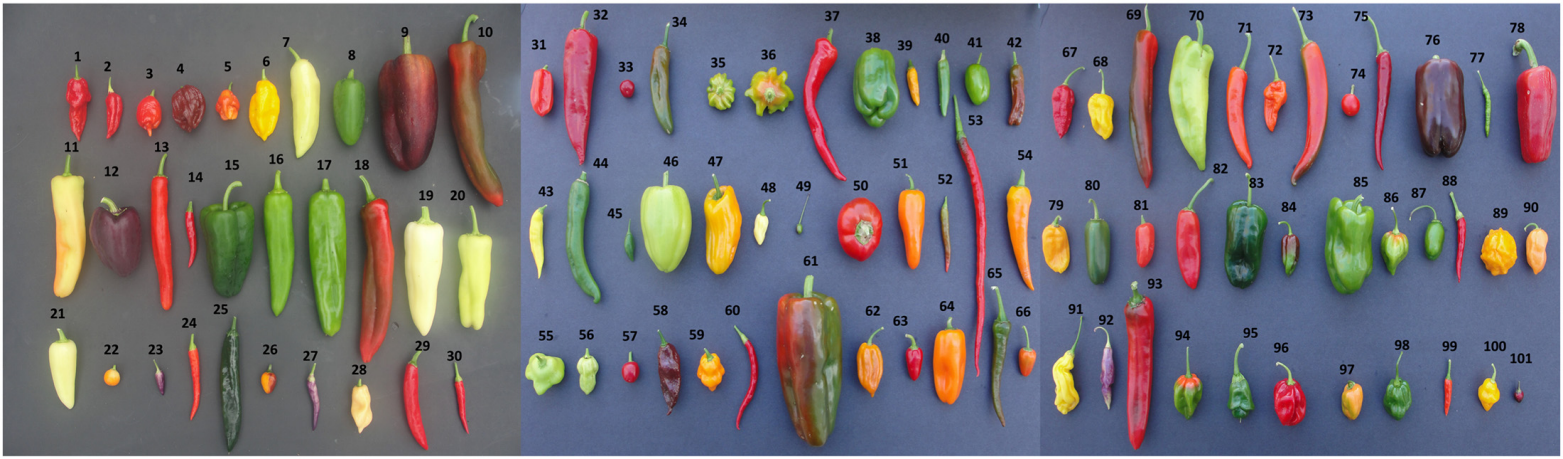
All Peppers explored for nutritional analysis. 1: Bhut Jolokia, 2: Trinidad 7 Pot, 3: Trinidad Butch T Scorpion, 4: Trinidad Douglah, 5: Trinidad Moruga Scorpion, 6: Yellow Bhut Jolokia (Ghost Pepper), 7: Aji Crystal, 8: Jalamundo, 9: Manzano, 10: Corno Di Toro, 11: Banana Sweet, 12: Lilac Bell, 13: Succette de Provence, 14: Thai Red, 15: Ancho, 16: Joe Parker, 17: Big Jim, 18: Sandia, 19: Feher Ozon Paprika, 20: Aconcagua, 21: Santa Fe Grande, 22: 5 Color Marble, 23: Twilight, 24: Naga Dorset, 25: Pasilla, 26: Gold Nugget, 27: Sangria, 28: Peach Habanero, 29: Korean, 30: Chilly Chili, 31: Scotch Bonnet, 32: Espanola, 33: Haiti Cluster, 34: Guajillo, 35: Atomic Starfish, 36: Nepal, 37: Spanish Cayenne, 38: Chinese Giant Sweet, 39: Amish Chicken, 40: Bulgarian, 41: Rocoto Yellow, 42: Shishito, 43: Aji Limon, 44: Sunrise, 45: Mayan Cobanero, 46: Szegedi Giant, 47: Marseilles Sweet Yellow, 48: White Habanero, 49: Tepin, 50: Apple Pimento, 51: Dulcetta Orange, 52: Japones, 53: Joe's Long Cayenne, 54: Tequila Sunrise, 55: Uba Tuba, 56: White Bhut Jolokia, 57: Lady Bug Cherry Bomb, 58: Brown Bhut Jolokia, 59: Orange Trinidad Moruga, 60: Chili De Arbol, 61: Big Bertha, 62: Mustard Habanero, 63: Bahama Fish, 64: Barancio Paprika, 65: India Byadagi Mirchi, 66: Gundo Mirchi, 67: Naga Morich, 68: Yellow Moruga, 69: Ancient Sweet, 70: Bever Dam, 71: Aji Amarillo, 72: Orange Suave, 73: Chinese Ching Choo, 74: Pakistan, 75: Paprika De Cayenne, 76: Sweet Chocolate Bell, 77: Orange Thai, 78: Buran, 79: Zavory Habanero, 80: Pinata, 81: Red Suave, 82: Piquillo, 83: Mulato, 84: Chocolate Habanero, 85: Marconi Gold Sweet Bell, 86: Red Habanero Hot, 87: Red Rocoto, 88: Laotian, 89: Jamaican Yellow, 90: Orange Habanero Hot, 91: Malaysian Goronong, 92: Cajamarca, 93: California Mild, 94: Antilles, 95: Peter Pepper Red, 96: Congo Red, 97: Pumpkin Habanero, 98: Golden Habanero, 99: Assam, 100: Trinidad Perfume, 101: Black Pearl.

Pepper types were sourced from various heirloom seed producers across North America (unless otherwise noted; S1 Table) to explore their phenotypic diversity. They come from multiple geographic origins, encompass both landraces (including heirlooms) and cultivars, and represent five different species (S1 Table). Seeds from each pepper type were sown in five gallon containers using a standard potting mix indoors under fluorescent lights followed by metal halide lighting. The soil was fertilized with Pure Blend Pro Grow (3-2-4), Pure Blend Pro Bloom (2-3-5), and Cal-Mag Plus (all from Botanicare, AZ) for a ten-week period following the manufacturer’s recommendations. Plants were then left in pots and grown outdoors in a completely random design with two replications in Madison, WI during the summer of 2013. Leaves and fruits were harvested in September 2013 (Fig 1). All ripe fruits were harvested from each plant.

### Fruit Tissue Preparation

A pepper from each pepper plant was washed under running water and dried off with paper towels. Five grams of fresh whole peppers, chosen randomly from each type, was cut lengthwise, then chopped into small pieces and put into 50 ml conical tubes. Eight stainless steel beads (SSB14B, 0.9–2.0 mm diameter) were added to each tube for homogenization with a bullet blender (tissue 50 homogenizer) for 12 min. 12.5 ml of 100% methanol (Sigma) (capsaicin and vitamin A assays), double distilled water adjusted to a pH 6.5 (folate assay), or Phosphate Buffered Saline (PBS) pH 7.0 (vitamin C assay) were added into the slurry and homogenized again for 12 min. The liquid portion of the final slurry was pipetted into a centrifuge tube, and centrifuged for 23 min at 4,000 rpm. The supernatant was filter-sterilized through a 0.2 μm Acrodisc filter and samples were stored at either -20°C (methanol extracts) or 4°C (water and PBS extracts) until used for each respective assay. This process was repeated for the second biological replicate with a different fruit from the same plant. One fruit from 101 pepper types was used for color analysis (Fig 1). Photographs were examined for quantitative color and shape using Tomato Analyzer 3.0 [37]. While 101 peppers were explored, vitamin assays were not successful for each pepper leading to fewer than 101 peppers in each univariate vitamin data set.

Vitamin A concentrations were estimated for 82 of the pepper types using a Vitamin A Food Enzyme-Linked Immunosorbent Assay (ELISA) technique following the manufacturer’s instructions (Crystal Chem Inc., IL) and a microplate reader (450 nm) (Epoch 2, Biotek). Vitamin C concentrations were estimated for 90 of the pepper types using an EnzyChrom^™^ Ascorbic Acid Assay Kit following the manufacturer’s instructions (BioAssay Systems, Hayward, CA) and a microplate reader (570 nm). Folate concentrations were estimated for 90 of the pepper types using a Folic Acid ELISA kit following the manufacturer’s instructions (Eagle Biosciences, Nashua, NH) and using a microplate reader (450 nm).

Capsaicin concentration was estimated for 90 of the pepper types using a Capsaicin HS Plate Kit (Beacon Analytical Systems Inc., Saco, ME) following manufacturer’s instructions and a microplate reader at 450 nm. The capsaicin content standard curve was developed using diluted amounts of pure capsaicin (Sigma). The capsaicin concentration was measured in ppm from the kit and multiplied by 16 to convert to SHU (Scoville Heat Units).

### Statistical analysis

Each univariate nutritional compound data set was analyzed using an Analysis of Variance with cultivar as a fixed factor on complete univariate data sets. Cultivar means for each compound were separated using a Fishers Least Significant Difference (LSD, α = 0.01) with a Bonferroni correction in the R statistical language and programming environment [38]. Pearson and Spearman correlation coefficients between nutritional compounds, shape, and color were tested with pepper types that had paired phenotypic information using R, this was done as an exploratory analysis due to the lack of replication in color measurements. Figures were made using R or Microsoft Excel.

### Exploring Public Data on Nutrition

Data from the USDA National Nutrient Database for Standard Reference Release 28 was downloaded to compare concentration of vitamin A, vitamin C, and Folate in the examined pepper types to other foods known to be high in the nutrients and the effect preparation techniques have on nutrient content.

## Results

**Fig 2:**
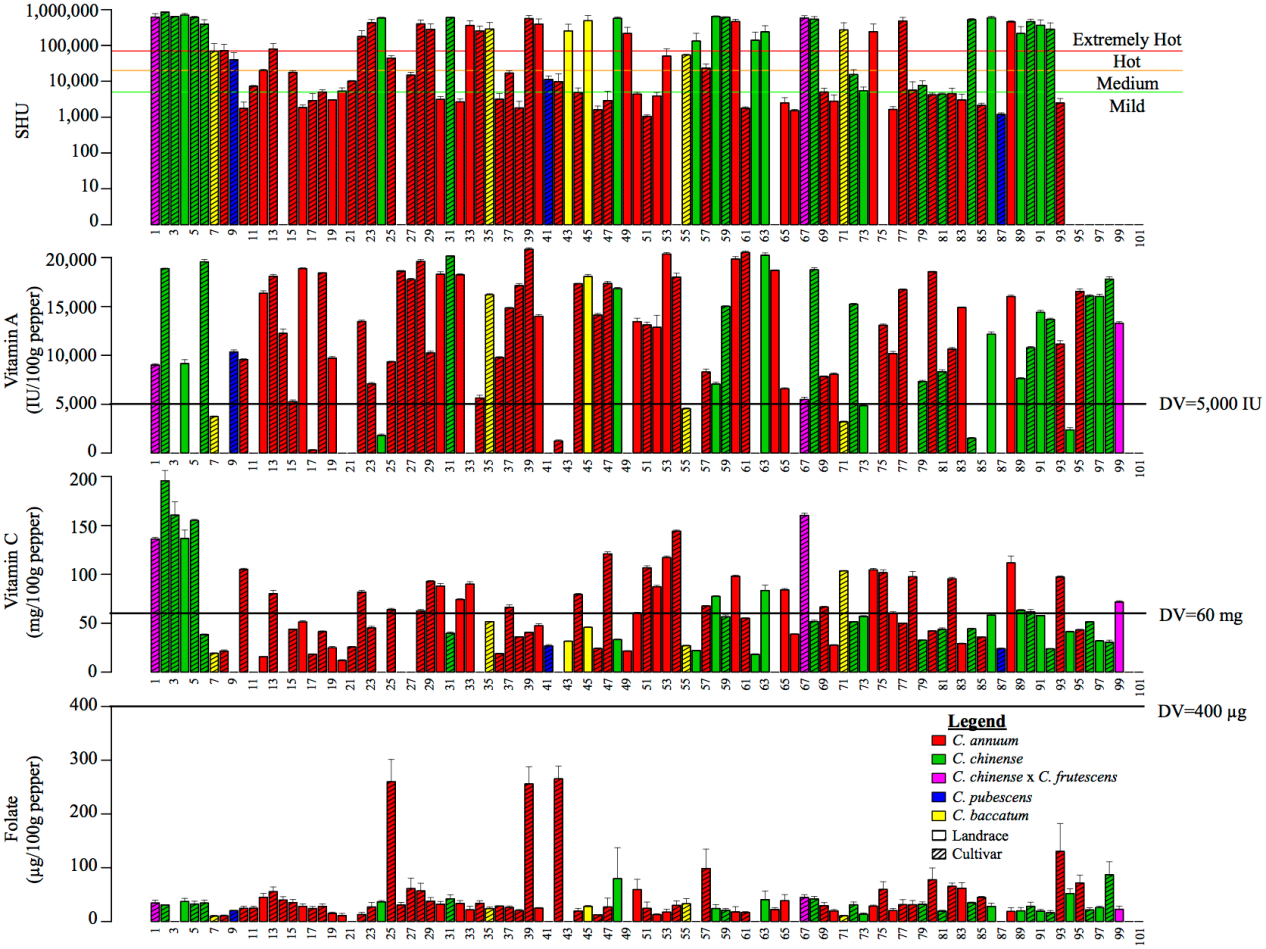
Levels of capsaicin (SHU), vitamin A, vitamin C, and folate detected in pepper types. In the plot, each bar represents a single pepper type in order of the numbering in Fig 1. Each bar represents the average of two biological replicates and the error bars correspond to standard error. The bars are colored based on species and shaded according to status as landrace or cultivar according to the legend. The top plot of capsaicin content is on a log base 10 scale with horizontal lines at 70,000 SHU (red), 20,000 SHU (orange), and 5,000 SHU (green) separating labeled levels on pungency. Recommended daily values (DV) for adults consuming 2,000 calories are plotted and labeled for the three lower plots of pepper nutritional content (FDA, 2011). LSD = 262963 SHU, 1624 vitamin A, 18.7 vitamin C, 94.3 folate.

**Fig 3:**
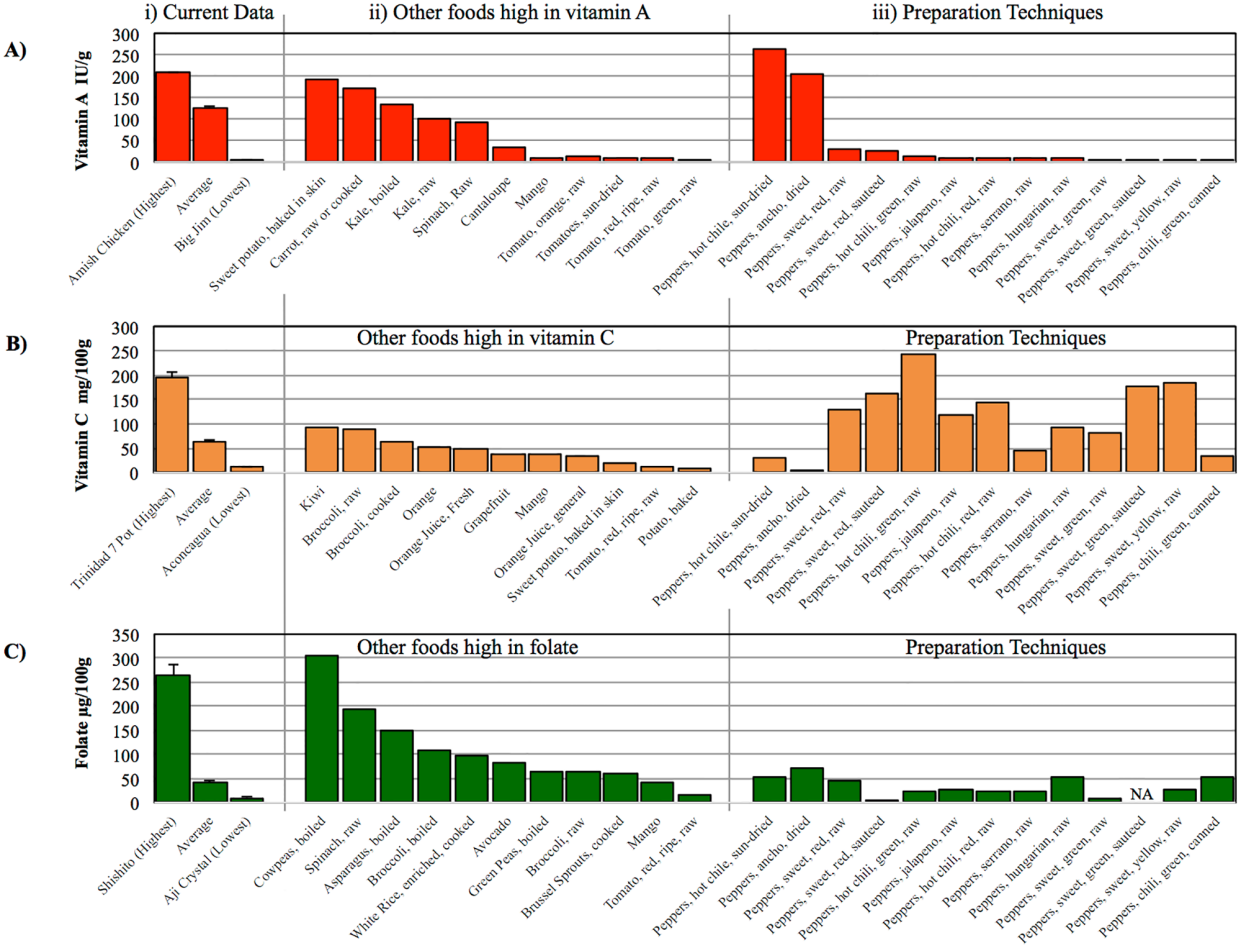
Comparison of our pepper types to other commonly consumed foods and preparation techniques. Plotted are the vitamin A (A), vitamin C (B), and folate (C) content for the current data (i), other foods high in each nutrient (ii), and preparation techniques from the USDA National Nutrient Database for Standard Reference Release 28. Error bars in the current data correspond to standard error.

The current collection showed a large diversity of shape, size, and color (Fig 1; raw color and shape data in S2 Table). These peppers ranged from sweet to among the hottest named types in the world and vitamin A, vitamin C, and folate content varied considerably (Fig 2; raw data for replicates in S3 Table). Capsaicin content, as estimated in SHU, ranged from mild (less than 5,000 SHU) to extremely hot (greater than 70,000 SHU) in the 90 types analyzed (Fig 2A). Vitamin A content ranged from 303 to 20,840 IU per 100 grams of pepper in the 82 types analyzed (Fig 2B). Vitamin C content ranged from 11.9 to 195.8 mg per 100 grams of pepper in the 90 types analyzed (Fig 2C). Folate content ranged from 10 to 265 micrograms per 100 grams of pepper in the 90 types analyzed (Fig 2D). Variation was continuous within the range identified for vitamin A and vitamin C, while, for capsaicin and folate, the distribution was more bimodal with cultivars either having very low or very high levels. The mean separations identified pepper types for each vitamin that were significantly different from each other (S3 Table). For each vitamin, many genotypes had similar values; however, 2–15% had significantly higher vitamin or heat content (S3 Table). The high concentration of vitamin A, vitamin C, or folate seen in individual pepper varieties was similar to other foods known to be high in these nutrients but would likely be affected by preparation technique (Fig 3). Capsaicin was positively correlated with Vitamin C, while other inter-factor relationships between capsaicin and nutrients content were limited (S4 Table).

On average in this sample, pepper types hailing from South America or Asia were hotter than those from North America (including Mexico, the origin of domestication) or Europe (S1 Fig). Peppers from Asia were also on average lower in vitamin A content than those from other continents. Status as a “cultivar” or “landrace” did not appear to be related to the factors measured. At the species level, *C*. *baccatum* and *C*. *chinense* were on average hotter than *C*. *annuum*. The average folate concentration was similar across species, but all of the high content outliers were *C*. *annuum*. While this sample size is limited, we can confirm that the range in nutrient content is high across species, regions, and cultivation status.

## Discussion

In this study, we observed high variation in nutrient content for peppers within species, variety, color, and geographic location. For 73 of these pepper types (89% of those assayed), a 100 gram serving of raw pepper (approximately equal to one jalapeno or half of a large bell pepper), exceeded the recommended daily value (DV) 5,000 IU of vitamin A set by the U.S. Food and Drug Administration for a 2,000 calorie diet in adults and children over age 4 [39]. A single serving of 39 of these pepper types (43% of those assayed) exceeded the 60 mg recommended DV of vitamin C [39]. A single serving of only three of these pepper types constitutes more than 50% of the 400 micrograms folic acid recommended DV [39]. Some of the pepper types we assayed had nutrient concentrations at or above levels observed in foods high in vitamin A, vitamin C, or folate on a per gram basis (Fig 3). Awareness of the high nutritional content in some of these varieties could benefit consumers, especially those in malnourished regions where peppers frequent the cuisine, and for plant breeders involved in improving peppers for multiple characteristics.

Vitamin A content has been shown to be highly variable between pepper varieties [13]; nevertheless, it was unexpected that many of our pepper types had higher levels of vitamin A than previously assayed varieties [13] and eight of our pepper types had higher vitamin A concentration (on a per gram basis) than sweet potato, which is often heralded for high vitamin A. The difference between vitamin A content in our pepper types and that measured in foods typically considered high in vitamin A shows great potential for improving nutrition both by increasing consumer awareness and improving genetics of peppers even in the US marketplace (Fig 3A). Further, there appears to be little reduction in vitamin A when peppers are raw, sautéed, or canned (Fig 3A), convenient for accommodating all preparation preferences. Amish Chicken (#39) was the pepper type highest in vitamin A, however it has an extremely hot flavor so little can be consumed at a time. Mild and medium heat pepper types like Big Bertha (#61), Joe’s Long Cayenne (#53), and Joe Parker (#16) have similarly high vitamin A and little heat, making them better candidates for pepper breeding programs or immediate dietary incorporation. Consumption of beta-carotene rich sweet potatoes improves vitamin A status in children [40]; perhaps consumption of pepper types high in vitamin A could be similarly effective (Fig 3A).

Peppers are well known for their high vitamin C content [12,21,41]. This was confirmed in our study where 16 pepper types had vitamin C concentration higher than that recorded for kiwi, a vitamin C rich fruit (Fig 3B). Our vitamin C estimates have a similar range to the pepper types noted in the USDA database (Fig 3B). While color data were not replicated, we did observe a positive correlation between vitamin C and pepper redness and a negative correlation with pepper greenness. This relationship between color and vitamin C content has been previously established [42,43]. Mature color in pepper is thought to be controlled by three loci [44,45]; these loci segregate both within and between the domesticated species. While not strongly related to nutritional compounds, color is very important to consumers [46]. Like vitamin A, the level of vitamin C in peppers is not reduced by sautéing; however, it is when peppers are dried (Fig 3B). Trinidad 7 Pot (#2) had the highest vitamin C content, but, again is extremely hot. The peppers with high vitamin C content and mild to medium flavor profiles, such as Joe’s Long Cayenne (#53), Marseilles Sweet Yellow Bell (#47), Dulcetta Orange (#51) and Corno Di Toro (#10), could prove useful in nutrition supplementation and breeding programs.

A few of the pepper types had very high folate levels (max 265.2 μg/100 g), higher even than raw spinach (193 μg/100 g; Fig 3C). This far exceeds previous estimates of up to 70 micrograms folic acid per 100 grams fresh pepper [11,41]. Currently mandated folic acid fortification practices in the US are estimated to provide 100–200 micrograms of folic acid per day to women of childbearing age [36], a similar level to that seen in 100 grams of Shishito (#42), Pasilla (#25), Amish Chicken (#39), or California Mild (#39). Folate content does seem to be affected by preparation techniques as sautéing peppers has been shown to drastically reduce folate content (Fig 3C). Breeding for increased folate content and being mindful of how peppers are prepared could prove useful in the continued global efforts to reduce the number of pregnancies affected by neural tube defects [36].

Capsaicin estimates for the extremely hot peppers were slightly lower than expected. For example, the hotness of Bhut Jolokia (#1) has been published at 1,001,304 SHU [47], whereas here it averaged 616,806 SHU, likely due to growing conditions, which can affect pungency [48,49].

Peppers show promise for breeding for nutritional content. The types assayed here that showed the most promise were different for each nutritional compound (Table 1, S2 Fig). Some cultivars, particularly Amish Chicken (#39), Trinidad 7 Pot (#2), Joe’s Long Cayenne (#53), Joe Parker (#16), and Big Bertha (#61), possessed positive values for several nutritional compounds (Table 1). These five types range in pungency from very hot (Amish Chicken (#39), Trinidad 7 Pot (#2)), to medium heat (Joe’s Long Cayenne (#53)) and mild (Joe Parker (#16) and Big Bertha (#61)). We found minimal correlations between nutritional phenotypes, so plant breeding with recurrent selection should produce cultivars high in all nutrients. Additionally, the minimal relationship between capsaicin content and vitamin content provides an opportunity to create cultivars with high nutritive content to fit the palate of any market. The high narrow-sense heritability of vitamin C [50] should support this goal and it will be important to calculate the heritability of the other nutrients. The finding that nutritional phenotypic variation occurs on multiple continents and in many species also means breeders should be able to work with local types rather than incorporate exotic germplasm to make nutritional gains. Conveniently, many of these pepper species are easily crossable (S5 Table)[51,52]. Thus, nutritious cultivars that also have the appropriate flavor and color profiles for diverse local markets should be attainable through breeding.

**Table 1:** Cultivars with highest and lowest nutrient content among those tested.

Capsaicin Content		Vitamin A Content		Vitamin C Content		Folate Content	
Ten Lowest	Ten Highest	Ten Lowest	Ten Highest	Ten Lowest	Ten Highest	Ten Lowest	Ten Highest
Dulcetta Orange	Trinidad 7 Pot	Big Jim^-^	Amish Chicken^+^	Aconcagua^-^	Trinidad 7 Pot^+^	Aji Crystal^-^	Shishito
Red Rocoto	Trinidad Douglah	Shishito	Big Bertha^+^	Lilac Bell	Trinidad Butch T Scorpion	Jalamundo^-^	Pasilla
Gundo Mirchi	Brown Bhut Jolokia	Chocolate Habanero	Joe's Long Cayenne^+^	Mustard Habanero	Naga Morich	Aji Amarillo^-^	Amish Chicken^+^
Szegedi Giant	Trinidad Butch T Scorpion	Naga Dorset^-^	Bahama Fish	Big Jim^-^	Trinidad Moruga Scorpion	Aconcagua^-^	California Mild
Sweet Chocolate Bell	Bhut Jolokia	Antilles	Scotch Bonnet	Nepal	Tequila Sunrise	Szegado	Lady Bug Cherry Bomb
Corno De Toro	Orange Trinidad Moruga	Aji Amarillo^-^	Chili De Arbol	Aji Crystal^-^	Trinidad Douglah	Japones	Golden Habanero
Big Bertha^+^	Trinidad Moruga Scorpion	Aji Crystal^-^	Peach Habanero	Jalamundo^-^	Bhut Jolokia	5-color marble	White Habanero
Chinese Giant Sweet	Scotch Bonnet	Uba Tuba	Yellow Ghost Pepper	Tepin	Marseilles Sweet Yellow Bell	Ching Choo	Pinata
Joe Parker^+^	Red Habanero Hot	Chinese Ching Choo	Joe Parker^+^	White Bhut Jolokia	Joe's Long Cayenne^+^	Feher Ozon	Peter Pepper Red
Marconi Gold	Naga Dorset^-^	Ancho	Trinidad 7 Pot^+^	Cajamarca	Laotian	Cajumarca	Piquillo

^+^ Pepper types that show up twice in this table for ten highest nutrient content or low capsaicin content.

^-^ Pepper types that show up twice in this table ten lowest nutrient content or high capsaicin content.

Direct consumption of the more nutritious peppers assayed here as well as future consumption of nutritionally enhanced varieties could be used in international efforts to address vitamin deficiency. Thus, though not a silver bullet, peppers could constitute an important part of an integrated strategy, including nutrient supplementation and food fortification, for combatting vitamin deficiency. While lower quantities of pepper are consumed daily compared to staples, such as maize or wheat, highly nutritious or nutritionally improved peppers can contribute to a diverse and healthy diet.

## Supporting Information

S1 Fig Boxplots of levels of capsaicin (SHU), vitamin A, vitamin C, and folate detected in pepper types based on continent of origin, cultivar or landrace, or species. The top plot of capsaicin content is on a log base 10 scale.
(TIFF)

S2 Fig A) Overlap of genotypes with the highest vitamin content and high capsaicin content. B) Overlap of genotypes with high vitamin content and low capsaicin content. The overlap between high SHU and vitamin C were the cultivars Bhut Jolokia, Trinidad Moruga Scorpion, Trinidad Butch T Scorpion, and Trinidad 7 Pot. The overlap between vitamin A and vitamin C was Joe’s Long Cayenne. The overlap between low SHU and vitamin A were Joe Parker and Big Bertha.
(TIFF)

S1 Table Seed Source for all pepper varieties used in this study. (XLSX)

S2 Table Color and shape data for peppers pictured in Fig 1 estimated with Tomato Analyzer 3.0. (XLSX)

S3 Table Raw values for nutritional compounds and capsaicin content. Evaluated by least significant difference for grouping, group critical values listed.
(XLSX)

S4 Table Pearson (upper right) and Spearman (lower left) correlations between chile pepper nutritional compounds, shape, and color components. Bold text indicates significant relationship (p<0.05); stronger red = more linear positive relationship; stronger blue = more linear negative relationship.
(XLSX)

S5 Table Crossing relationships of Capsicum cultivated species relative to each other and wild species. (XLSX)
