# Supplementary figures and images for: Vitamin Variation in Capsicum Spp. Provides Opportunities to Improve Nutritional Value of Human Diets
Source: PLoS One. 2016 Aug 17;11(8):e0161464. doi: 10.1371/journal.pone.0161464 (PMC4988645; doi:10.1371/journal.pone.0161464)

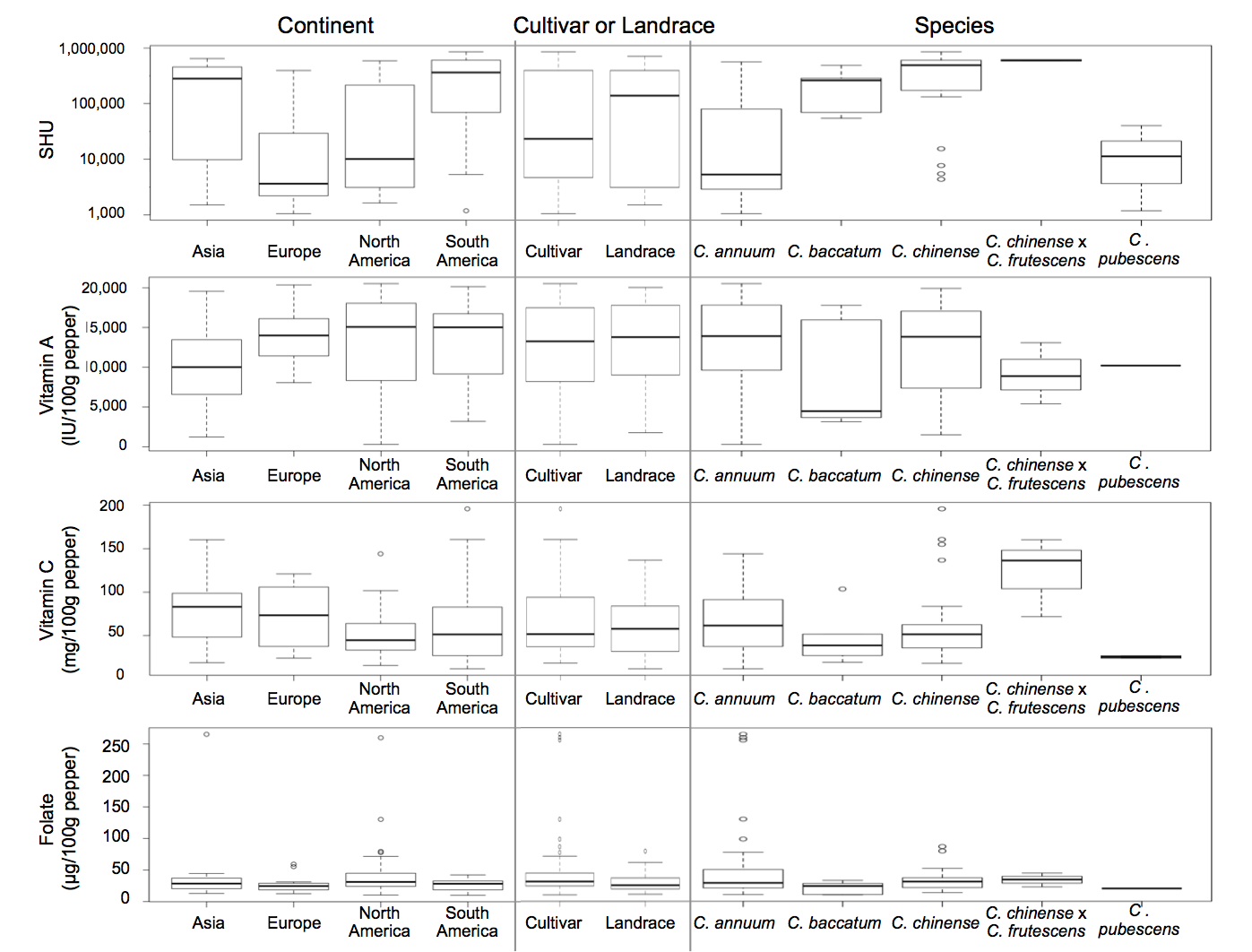

Supplement: S1 Fig — The top plot of capsaicin content is on a log base 10 scale. (TIFF) [file pone.0161464.s001.tiff]

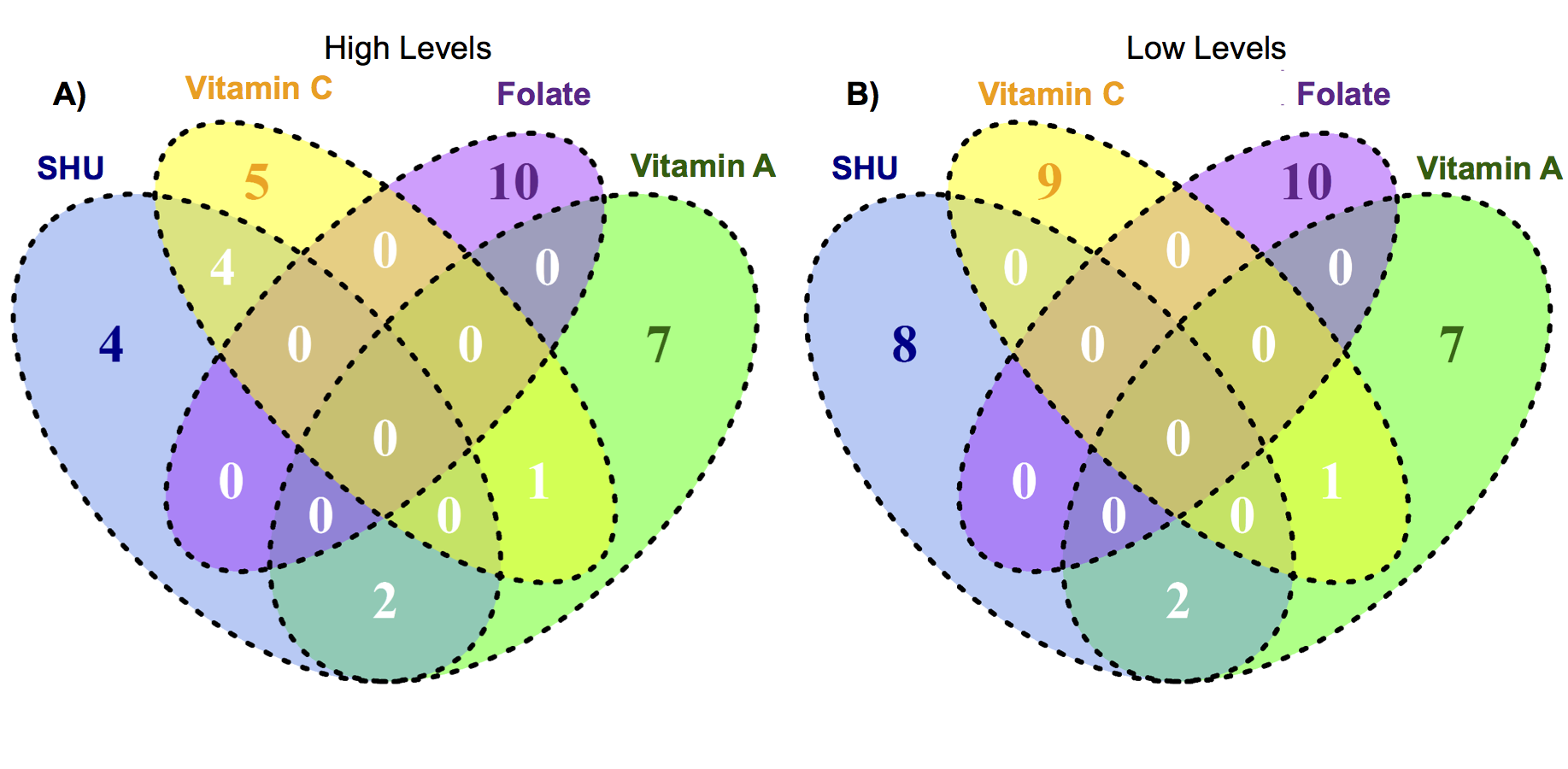

Supplement: S2 Fig — A) Overlap of genotypes with the highest vitamin content and high capsaicin content. B) Overlap of genotypes with high vitamin content and low capsaicin content. The overlap between high SHU and vitamin C were the cultivars Bhut Jolokia, Trinidad Moruga Scorpion, Trinidad Butch T Scorpion, and Trinidad 7 Pot. The overlap between vitamin A and vitamin C was Joe’s Long Cayenne. The overlap between low SHU and vitamin A were Joe Parker and Big Bertha. (TIFF) [file pone.0161464.s002.tiff]
